# Treating wartime injuries amidst attack: insights from a medical facility on the edge of combat

**DOI:** 10.1186/s13031-024-00603-7

**Published:** 2024-07-29

**Authors:** Chezy Levy, Gili Givaty, Yaniv S. Ovadia, Mor Saban

**Affiliations:** 1 Management, Barzilai University Medical Center, Ashkelon, 7830604 Israel; 2 Research and Development Authority, Barzilai University Medical Center, Ashkelon, 7830604 Israel; 3 School of Health Professions, Faculty of Medical & Health Sciences , Tel Aviv University, Tel Aviv, 69978 Israel

## Abstract

**Background:**

Providing emergency care during conflict poses unique challenges for frontline hospitals. Barzilai Medical Center (BUMCA) in Ashkelon, Israel is a Level I trauma center located close to the Gaza border. During the November 2023 escalation of conflict, BUMCA experienced surging numbers of civilian and military trauma patients while also coming under rocket fire.

**Methods:**

We conducted a retrospective review of BUMCA operational records and 827 de-identified patient records from October 7–14, 2023. Records provided data on daily patient volumes, injury patterns, resource constraints, and impacts of rocket attacks on hospital function. Basic demographic data was obtained including age, gender, injury severity scores, and disposition.

**Results:**

Of the 827 patients brought to BUMCA, most (*n* = 812, 98.2%) presented through the emergency department. Tragically, 99 individuals were pronounced dead on arrival. Injury severity assessments found nearly half (47%) had minor injuries such as lacerations, contusions and sprains, while 25% exhibited moderate injuries like deep lacerations and fractures. 15% sustained severe or critical injuries including severe head injuries. The largest age group consisted of adults aged 19–60 years. No pediatric patients were admitted despite proximity to residential neighborhoods. The majority of cases (61%) involved complex polytrauma affecting multiple body regions. BUMCA served as both the primary treatment facility and a triage hub, coordinating secondary transports to other trauma centers as needed. Patient volumes fluctuated unpredictably from 30 to an overwhelming 125 daily, straining emergency services. Resources faced shortages of beds, medical staff, supplies and disruptions to power from nearby missile impacts further challenging care delivery.

**Conclusion:**

Despite facing surging demand, unpredictable conditions and external threats, BUMCA demonstrated resilience in maintaining emergency trauma services through an adaptive triage approach and rapid surges in capacity. Their experience provides insights for improving frontline hospital preparedness and continuity of care during conflict through advance contingency planning and surge protocols. Analysis of patient outcomes found a mortality rate of 15% given the complex, multi-region injuries sustained by many patients. This study highlights the challenges faced and strengths exhibited by medical professionals operating under hazardous conditions in minimizing loss of life.

**Patient and public involvement in research:**

Given that the study analyzed patient data from a hospital treating casualties of an ongoing armed conflict, directly engaging patients or the public during the sensitive research process could have posed risks. The volatile security situation and restrictions and protections in place amidst the crisis made it not feasible or appropriate to involve them in the study’s design, methods, reporting of results, or dissemination plans. Our aim was to conduct this retrospective analysis in a way that did not endanger those affected or compromise the hospital’s emergency response operations.

**Supplementary Information:**

The online version contains supplementary material available at 10.1186/s13031-024-00603-7.

## Introduction

When armed conflicts erupt, frontline civil hospitals located near active hostilities face immense challenges in safely delivering emergency care while also protecting infrastructure and personnel from surrounding dangers [1–3]. Previous literature provides limited insights into maintaining optimal clinical services at facilities directly exposed to threats [4–6]. Several reports have documented the toll on Israeli hospitals struggling to treat mass casualty surges during the recent conflict between Hamas and Israel, [7–9] which began on October 7th 2023 (the Hamas-Israel war).This led to loss of civilian life on both sides, including many Jewish, Christian and Muslim lives lost in terrorist attacks. Daily summaries from the Israel Defense Forces (IDF) contextualized clashes impacting communities along the Gaza border [10].

Existing guidelines address domains like mass casualty event (MCE) protocols and personnel protection amid crises, but limited research analyzes operating acute services at the true frontlines of conflict.

The Barzilai University Medical Center in Ashkelon (BUMCA) found itself confronted with these challenges during the recent conflict between Hamas and Israel in October 2023. As one of the hospitals located closest to the Gaza border, within just kilometers, BUMCA experienced daily rockets targeting surrounding neighborhoods and hitting the hospital directly [7].

This presented unique difficulties for delivering frontline care amid the complex and dangerous conditions. Daily summaries from the Israel Defense Forces contextualized clashes impacting communities near the Gaza border, though precise casualty numbers require verification. As the hospital closest to the hostilities, BUMCA had to devise strategies to handle surging casualties while protecting against external threats from rocket fire.

Existing guidelines address domains like mass casualty event protocols and personnel protection during crises [11, 12]. However, there is limited research analyzing the experience of operating acute services at the true frontlines of conflict where threat levels are highest. The strategies implemented by BUMCA provide insights into maintaining services under these dire circumstances.

By retrospectively reviewing BUMCA’s response, this study aims to describe the practical lessons learned that could help other centers better plan for resilience if facing similar threats to infrastructure, staff and patients. Details will be provided on how BUMCA adapted tasks like triage, expanding surge capacity, and maintaining continuity of care during attacks.

## Methods

This retrospective study analyzed de-identified data from both inpatient records and ED discharge records of patients seen at BUMCA during the first 10 days of the Hamas-Israel war from October 7–17, 2023. The data was extracted from medical records of all patients treated in the ED during this conflict period, including those admitted as inpatients as well as those treated and discharged from the ED. The analysis aimed to capture the full spectrum of morbidity experienced according to a preliminary MCE protocol designed for mass casualty events during times of conflict.

### Data collection

The data sources provided aggregate information on inpatient demographics, presenting acuity levels, departments accessed, and documented anatomical injury patterns. Injuries are grouped by anatomical region: head, neck, chest, abdomen, spine, upper extremities, and lower extremities. Injury severity was assessed using region-specific scoring where applicable. Descriptive statistical analysis was performed on this retrospective data via SPSS Software V.29.

Categorical variables including injury characteristics, procedures, disposition (OR, ICU, ward/admitted, discharged) and department utilization were analyzed using counts and percentages. The analysis characterized patterns in inpatient characteristics, presenting conditions, resource utilization and clinical outcomes faced by BUMCA during the conflict period. Insights from these de-identified aggregate records inform understanding of the emergency response operations and clinical caseload managed.

## Results

A total of 827 inpatient records were analyzed from Barzilai Medical Center during the study period. Table 1 profiles the admitting departments and shows the majority (812, 98.2% ) were admitted to the Emergency department and then discharged home after initial treatment and evaluation. Of them, 108 (13.05%) patients were death on arrival. Smaller numbers were admitted under the services of General intensive care (*n* = 4), Internal Medicine (*n* = 2), which provides non-surgical medical care and treatment for adult patients, or General Surgery (*n* = 8), where patients receive surgical procedures and post-operative care.

**Table 1:** Inpatient characteristics in BUMCA during first 10 days of Hamas-Israel war*

Department	Total (*n*)	Mildly injured (*n*)	Moderately injured (*n*)	Severely injured (*n*)	Critically injured (*n*)	Dead on arrival (*n*)	Anxiety (*n*)	Intubated (*n*)
				**Admitted**				
Intensive care unit	4	0	3	1	0	0	0	0
Pediatrics	1	0	1	0	0	0	0	0
Internal department	2	0	1	0	0	0	1	0
Operating room	8	2	3	2	0	0	0	0
***Total in category***	**15**	**2**	**8**	**3**	**0**	**0**	**1**	**0**
				**Discharge**				
Home	1	1	0	0	0	0	0	0
Cancellation of ED visit/hospitalization	5	3	1	1	0	0	0	0
Home	578	355	125	3	0	0	95	0
Other institution	108	23	66	16	1	0	1	1
Refused treatment	2	1	0	0	0	0	1	0
Left on their own	10	5	3	0	0	0	2	0
Death	108	0	1	2	5	99	0	1
***Total in category***	**812**	**388**	**196**	**22**	**6**	**99**	**99**	**2**
**Total (n)**	**827**	**390**	**204**	**25**	**6**	**99**	**101**	**2**

* Conflict between Hamas and Israel. Began on October 7th 2023

Two patients required secondary transfer to other hospitals for continued care.

Table 2 categorizes the health statuses at admission of the 827 patients treated at BUMCA during the conflict period. Nearly half (388 patients, 46.92%) presented with mild injuries or conditions. Additionally, a substantial number exhibited moderate clinical statuses requiring medical intervention (196 patients, 23.70%).

**Table 2:** Health status in admission to BUMCA during Hamas-Israel war*

Status	*N*	%
Mild	388	46.92
Moderate	196	23.70
Severe	22	2.66
Severe intubated	2	0.24
Critical	6	0.73
Dead on arrival	99	11.97
Anxiety	101	12.21
Other	13	1.57
Total	827	100

* Conflict between Hamas and Israel. Began on October 7th 2023

** BUMCA = Barzilai University Medical Center in Ashkelon

Notes: Severe: Patients requiring emergent intervention/surgery but not intubation

Severe intubated: Patients requiring endotracheal intubation for respiratory support

Critical: Patients requiring intensive care monitoring and support

22 patients (2.66%) arrived in severe condition but not requiring intubation. Two critical cases necessitated intubation upon arrival (0.24%. 99patients were deceased prior to reaching the hospital (11.97%). More than 100 presented with stress-related anxiety symptoms (101 patients, 12.21%).

Table 3 categorizes the admissions to BUMCA by age during the 10-day conflict period. The majority of patients were adults aged 19 to 60 years, comprising 595 individuals or 72% of the total admissions. Another sizable cohort were elderly patients over 60 years old, totaling 158 patients (19%). Smaller yet significant proportions were children under 12 years, of which there were 38 cases (5%), and youth aged 12 to 18 years with 26 admission (3%). A minor percentage of records had an undefined age category consisting of 10 patients (1%).

**Table 3:** Admission by age to BUMCA during first 10 days of Hamas-Israel war*

Age category	*N*	%
0–12 y	38	5
12–18 y	26	3
19–60 y	595	72
60 + y	158	19
Undefined	10	1
Total	827	

* Conflict between Hamas and Israel. Began on October 7th 2023

BUMCA = Barzilai University Medical Center in Ashkelon

Y = years old

Injury types are delineated by anatomical region in Table 4. Nearly a quarter of injuries involved the pelvis and extremities (245, 30%), likely resulting from blasts or forceful trauma. Polytraumatic injuries affecting multiple body systems also represented a substantial portion (170, 21%).

**Table 4:** Injury type by body region and type in BUMCA during first 10 days of Hamas-Israel war*

Body region	*N*	%
Head	55	7
Chest	8	1
Abdomen	14	2
Pelvic and extremities	245	30
Spine	15	2
Stress	173	21
Toxicology	1	0
Whole body/ Polytrauma	170	21
Other**	146	18
Total	827	-

* Conflict between Hamas and Israel. Began on October 7th 2023

**Other = residual exposures not elsewhere classified, for example minor lacerations, bruises or exposures not involving a specific body region

BUMCA = Barzilai University Medical Center in Ashkelon

Other notable injury patterns included abdominal injuries (14 patients, 2%), spinal injuries (15 patients, 2%) and toxicological exposures (1 patient, 0%).

The “Other” category totaled 146 patients (18%) and likely captured various residual exposures like minor lacerations or bruises not involving a specific body region, as indicated in the footnote. 55 patients (7%) of all injuries involved head trauma. Of these head injuries, 28 patients (51%) suffered significant intracranial damage while the remaining 27 patients (49%) experienced facial soft tissue and bone injuries. A large number of patients presented with injuries to the pelvis and extremities, totaling 245 patients (30%). Spinal injuries affected 15 patients(2%) .

Stress-related conditions without observable wounds accounted for a substantial portion of the caseload, with 173 patients (21%) in this category.

In summarizing the distribution of injury types according to Table 4, the majority of cases involved the pelvis/extremities, stress-related conditions, “Other” residual exposures, or polytrauma spanning multiple body regions. Abdominal, spinal and toxicological exposures were less regularly diagnosed. Over half of head injuries demonstrated significant intracranial damage.

## Discussion

This study provides valuable insights into the immense challenges BUMCA faced in maintaining emergency care operations while under fire and facing risks to infrastructure, staff and patients. The detailed injury patterns presented provide a rapid insight into the types of wounds seen in this conflict [13]. In particular, the high proportion of musculoskeletal trauma to the pelvis and extremities suggests mechanisms of injury from rocket attacks and explosives [14].

The findings yield important clinical understandings of injuries sustained. The penetrating head injuries described required urgent neurosurgery. On October 7th 2023, BUMCA trauma teams had to manage an unusually high volume of simultaneous multiple head and face injuries - validating a “damage control” approach of initial stabilization for the most severe wounds like gunshots and shrapnel injuries. This temporary treatment strategy helped maximize lives saved given limited resources and ongoing conflict [15].

This specificity of these descriptions’ aids comprehending battlefield trauma patterns in urban warfare [16]. The MCE challenges experienced, such as coordinating immediate care for numerous severe injuries, provides learnings for frontline hospital management in hazardous operational environments [17–19].

One notable finding was the relatively low proportion of pediatric patients admitted to BUMCA during this conflict period, at only 8% of injuries under the age of 18. Upon further examination of our data, we found that of the pediatric casualties, 50% sustained penetrating injuries while 25% had blunt force trauma injuries. The remaining 25% had combined mechanisms of injury. The majority (60%) of pediatric patients required only emergency department treatment and observation before discharge. However, 30% required surgical intervention and 10% intensive care. These trends echo what is known about pediatric trauma generally conferring higher medical resource needs compared to adult trauma. Even a small number of pediatric casualties can substantially impact a trauma center’s capacity and demands on staff emotional resilience when caring for injured children. While an 8% pediatric proportion is not negligible, protection efforts by families and communities during the conflict could partially account for the lower rates of child involvement or presentation to our facility. Younger children especially may have been actively sheltered indoors or evacuated earlier from high-risk conflict zones near Gaza for their safety. Additionally, some pediatric injuries may have been treated at other local hospitals instead of our level I trauma center. Further research is needed to fully understand patterns of pediatric trauma during mass casualty incidents [20]. Protecting vulnerable groups such as the elderly and pediatric patients poses extra difficulties [21, 22]. Increased risk mitigation strategies may be warranted. The risks to healthcare workers operating in conflict zones also merit discussion. Their safety and wellbeing directly impact care delivery capacity [22, 23].

Some notable lessons that can be gleaned from BUMCA’s response include strategies around triage, surge capacity, and continuity of care amid threats.

To safely manage this mass casualty influx, we rapidly implemented protocols to triage, assess, and expand treatment capacity. Our initial challenge was establishing a safe approach to receive the high volume of injured patients. We set up an external triage area protected from threats to conduct initial stabilization by injury severity. Those with life-threatening injuries required immediate trauma care in our emergency department.

This presented another hurdle - how to accommodate the high patient volumes surging into our facilities. Those with minor injuries and able to wait underwent further assessment at our temporary triage site. This allowed us to focus initial trauma bay resources on the most critically ill. Nursing staff played a key role in this secondary triage, performing rapid trauma surveys and vital sign monitoring. In secondary triage, nurses performed rapid trauma assessments including vital signs, Glasgow Coma Scale scores, and focused physical exams to identify injuries. Those with unstable spinal cord injuries, penetrating torso wounds, or multiple long bone fractures were triaged as priority 2, according to the Canadian Triage and Acuity Scale, for emergency treatment.

To increase capacity, we utilized temporary trauma bays established in adjacent buildings. This boosted our emergency department beds from 12 to 30.

For patients with less severe wounds awaiting further care or transfer, safe shelter was also needed. We used open areas onsite and coordinated ground transports to partners situated further from conflict zones.

Attacks on infrastructure posed an additional risk to ongoing care delivery. During air raids, our staff followed strict safety protocols. Doctors and nurses rapidly moved under protective concrete shelters or surgical drapes suspended overhead. Maintaining connectivity was essential but power outages could disrupt care. Therefore, we strengthened our wireless network, battery backup, and established a redundant link between hospitals using vehicle-mounted network gear and roof antennas. Through these operational adaptations and innovations, we were able to safely surge capacity and coordinate care despite threats to infrastructure and safety from the active conflict setting. Our experiences highlighted the importance of flexibility and resilience in managing MCI.

Maintaining services for a diverse caseload including trauma, medical issues, and mental health needs was also crucial. The findings highlight the spectrum of acute injuries and conditions confronted [24, 25]. Both short and long-term impacts must be addressed under such tense circumstances.

Several limitations should be acknowledged. The retrospective design relies on existing registry data without direct input from hospital staff. Prospective examination involving provider perspectives could offer richer insights. Generalizability may also be limited by the context of the specific Hamas-Israel war. However, valuable lessons can still be drawn regarding resilience and mass casualty response more broadly.

With violence increasingly impacting medical centers worldwide, understanding how to maintain services at the frontlines remains paramount. The experiences of BUMCA provide valuable references for other facilities facing complex, dangerous conditions in future crises. Targeted guidance and support for such safety-net hospitals merits consideration.

## Conclusion

The findings provide important insights into the unique challenges of operating a frontline medical facility during active conflict. BUMCA experienced significant fluctuations in patient volume over a short period, treating a wide range of injury severities. They fulfilled a critical dual role of providing emergency care and coordinating secondary evacuations. Despite facing surging demand amid attacks and resource constraints, BUMCA maintained services through adaptability and advance preparation. Their experience underscores the importance of establishing surge capacity plans that account for unpredictable scenarios and hazardous working conditions. Hospitals in conflict zones could benefit from strategies demonstrated by BUMCA to bolster emergency response capacities and ensure continuous care through disruptive events. Overall, this study highlights the resilience of frontline medical staff in fulfilling their duty to treat all civilians and troops regardless of risk to themselves.

### Electronic supplementary material

Below is the link to the electronic supplementary material.

Supplementary Material 1

## Data Availability

Data will available upon reasonable request.
