# Supplementary material for: Treating wartime injuries amidst attack: insights from a medical facility on the edge of combat
Source: Confl Health. 2024 Jul 29;18:47. doi: 10.1186/s13031-024-00603-7 (PMC11285414; doi:10.1186/s13031-024-00603-7)
Supplement: Supplementary file 1 — Supplementary Material 1 [file 13031_2024_603_MOESM1_ESM.docx]

**Supplemental files** - Map of Israel southern coastal area, showing BUMCA's proximity to the Gaza strip and its surroundings.


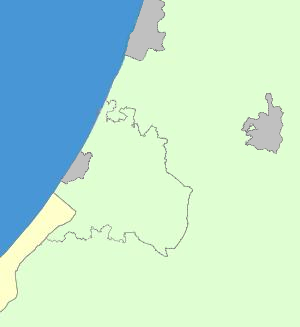


**BUMCA → •**

Gaza

Strip

Jerusalem

City

Ashkelon

sub-district

Ashkelon

City

***Mediterranean***

***Sea***

Tel-Aviv

Metropolis

*Reproduced with permission from The Israel Central Bureau of Statistics: http://gis.cbs.gov.il/benyam/
